# A chromosome-level reference genome assembly of the King Ratsnake (*Elaphe carinata*)

**DOI:** 10.1038/s41597-026-07692-4

**Published:** 2026-07-02

**Authors:** Boyang Liu, Liangyu Cui, Shangchen Yang, Jiayi Li, Ruyi Huang, Yihang Fan, Jin Chen, Chen Lin, Xiaojun Liu, Wenjing Sun, Haimeng Li, Yanling Xia, Yuan Fu, Song Huang

**Affiliations:** 1 College of Wildlife and Protected Area, Northeast Forestry University, Harbin, 150040 China; 2 College of Life Sciences, Zhejiang University, Hangzhou, 310058 China; 3 Shanghai Universities Key Laboratory of Marine Animal Taxonomy and Evolution, Shanghai Ocean University, Shanghai, 201306 China; 4 Anhui Province Key Laboratory of the Conservation and Exploitation of Biological Resource, College of Life Sciences, Anhui Normal University, Wuhu, 241000 China

## Abstract

The King Ratsnake (*Elaphe carinata*), a widely distributed non-colubrine snake in East Asia, exhibits a remarkable capacity for broad-spectrum venom resistance, enabling it to prey on other snakes, including lethal viperid and elapid species. Despite its ecological and physiological significance, the genetic basis of this trait remains largely unknown. Here, we present a chromosome-level genome assembly of *E. carinata*, generated using PacBio HiFi long-read sequencing, Illumina short-read data, and RNA-seq—supported by a synteny-based scaffolding approach using the closely related *Elaphe schrenckii*. The 1.62 Gb assembly (contig N50 = 2.77 Mb, scaffold N50 = 143.07 Mb) achieves 97.3% BUSCO completeness, with over 90% of sequences anchored into 18 pseudochromosomes. Repetitive sequences account for 53.19% of the genome, with LINE elements being the most abundant. We annotated 19,750 protein-coding genes, of which 99.2% were functionally assigned using integrated evidence from homology, transcriptomics, and ab initio predictions. This high-quality genomic resource provides a foundation for exploring colubrid evolution, venom resistance mechanisms, and the development of novel antivenom therapies.

## Background & Summary

The King Ratsnake is a large oviparous species belonging to the family Colubridae. Phylogenetically, it is classified within the non-colubrine lineage of colubrid snakes. This species is widely distributed across southeastern China, from northern Guangdong, Guangxi, and Taiwan in the south to the Beijing-Tianjin region in the north, with its range also extending into northern Vietnam and parts of Japan^1,2^. It predominantly inhabits montane and hilly areas, serving as an ecologically representative colubrid species. Its distribution pattern provides valuable insights into the biogeographic evolution of snakes from temperate to subtropical regions in East Asia.

*E. carinata* primarily preys on rodents, birds, and bird eggs, with notable ontogenetic variation in body coloration and size^3^. When threatened, it exhibits a distinctive defensive behavior by secreting a malodorous liquid from its cloacal glands. Notably, *E. carinat*a exhibits ophiophagous behavior and has evolved specific blood proteins that confer resistance to a spectrum of venomous snakes^4^. Its serum demonstrates an ability to neutralize hemorrhagic toxins from pit vipers, such as *Trimeresurus stejnegeri* and *Deinagkistrodon acutus*, as well as the potent neurotoxins from elapids, including the highly lethal *Bungarus multicinctus*^5–7^. Although these broad-spectrum antitoxic properties have been experimentally validated, the precise molecular mechanisms governing this immune protection and toxin metabolism remain elusive. A high-quality reference genome for this species would therefore provide a critical foundation for identifying the genetic basis of venom resistance and for advancing our understanding of the molecular adaptations underlying this remarkable trait.

In this study, we present the first chromosome-level genome assembly for the King Ratsnake, generated by integrating PacBio HiFi long-read sequencing, whole-genome sequencing (WGS) and RNA-seq data. Leveraging a reference-guided strategy based on a closely related species, this high-quality genomic resource provides a foundation for investigating colubrid evolution, chromosomal rearrangements, and population genetics. As a key resource, it will empower future studies aimed at elucidating the genetic basis of the species’ exceptional venom resistance, with potential implications for developing novel antivenom strategies and biomedical applications.

## Methods

### Sample collection and ethics statements

Muscle tissue was collected from a single adult female *E. carinata* found in Anhui Province, China, for DNA extraction, Mixed tissues from the liver, heart, kidney, and muscle were collected for RNA extraction. The sample was immediately flash-frozen in liquid nitrogen and stored at −80 °C until DNA and RNA extraction. The study design, experiments, and sample collection were approved by the Animal Ethics Committee of Northeast Forestry University (No.2023048).

### DNA & RNA extraction, libraries preparation and sequencing

High-molecular-weight genomic DNA was isolated from approximately 50 mg of muscle tissue using a QIAGEN DNeasy Blood & Tissue Kit. The integrity of the extracted DNA was verified by 1% agarose gel electrophoresis, and its concentration was quantified using a NanoDrop One UV-Vis spectrophotometer (Thermo Fisher Scientific, USA) and a Qubit 4.0 Fluorometer (Invitrogen, USA). The DNA was aliquoted for two distinct sequencing applications: one aliquot was used to construct a PacBio HiFi library with the SMRTbell Express Template Prep Kit 2.0 and sequenced on the PacBio Sequel IIe platform; another aliquot was used to prepare a short-insert library for whole-genome sequencing (WGS) on the Illumina platform with 100 bp paired-end reads.

Total RNA was extracted from a mixture of different tissues using TRIzol reagent (TIANGEN) according to the manufacturer’s protocol. Poly(A) RNA was enriched from total RNA using the Dynabeads mRNA Purification Kit (Cat#61006, Invitrogen) and fragmented using the fragmentation reagents included in the MGIEasy RNA Library Preparation Kit V3.1 (Cat#1000005276, MGI). First-strand cDNA was synthesized using random primers and reverse transcriptase, followed by second-strand synthesis. To generate strand-specific libraries, dUTP was substituted for dTTP during second-strand synthesis, and the subsequent PCR amplification was performed using a polymerase that does not amplify uracil-containing templates, thereby retaining strand orientation. The double-stranded cDNA was subjected to end repair, A-tailing, and adapter ligation. Size selection was performed using MGIEasy DNA Clean Beads (Cat#1000005279, MGI), followed by PCR amplification. For sequencing, double-stranded libraries were denatured to single-stranded DNA, and circularization was performed using the MGIEasy Circularization Module (Cat#1000005260, MGI). The resulting single-stranded circular DNA libraries were sequenced on the DNBSEQ-T7RS platform with 100 bp paired-end reads.

Finally, we generated a total of approximately 138.5 Gb of sequencing data, including 23 Gb of PacBio HiFi reads, 103 Gb of whole-genome shotgun (WGS) reads, and 12.5 Gb of RNA-seq reads.

### Genome assembly and assessment

**Fig. 1:**
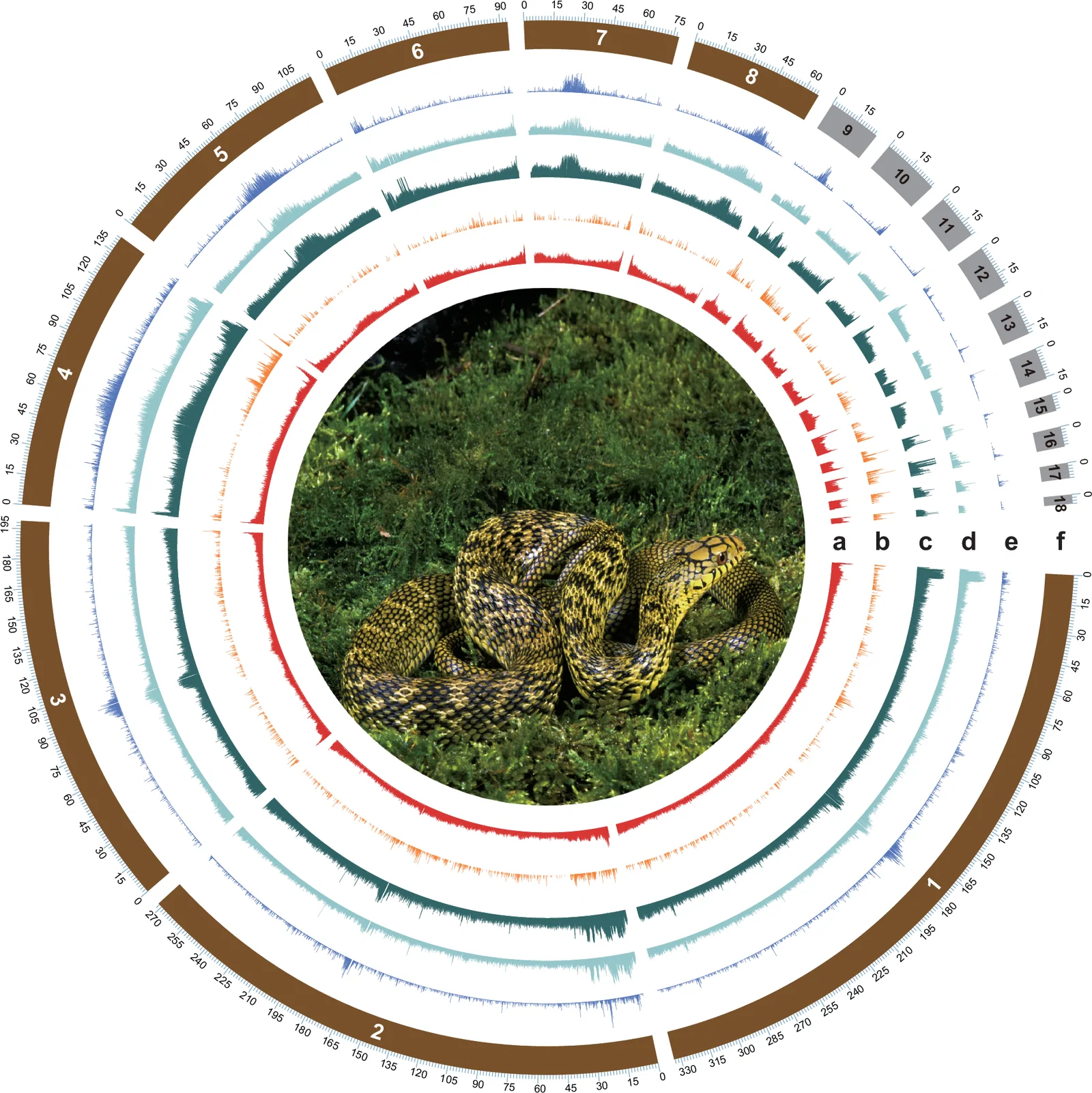
Genomic landscapes of the King Ratsnake (*E. carinata*). (a) GC ratio; (b) gene density; (c) TE density; (d) LINE density; (**e**) LTR density; (**f**) chromosome length (brown, macrochromosome; grey, microchromosome). The photo of a King Ratsnake was taken in the field (photo from Alamy).

**Fig. 2:**
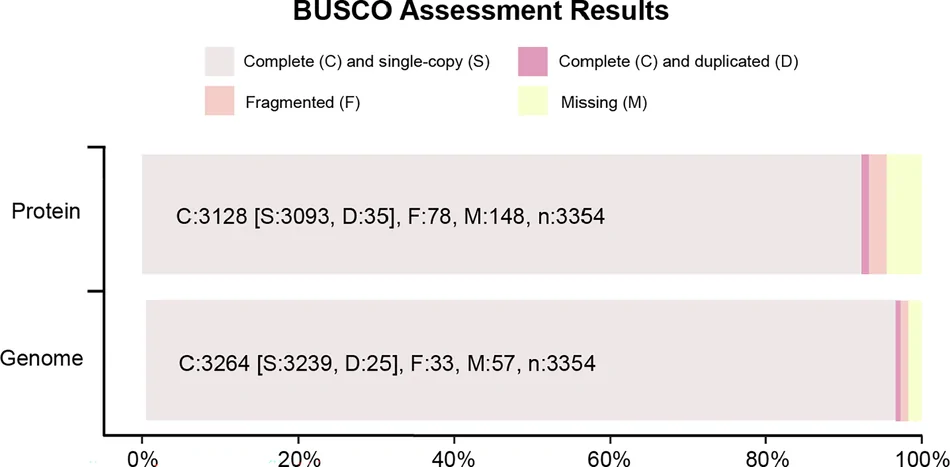
BUSCO assessment of *E. carinata* genome completeness.

The de novo genome assembly of *E. carinata* was generated using PacBio HiFi long-read data. Initial contigs were assembled with hifiasm (v0.19.5)^8^ and polished using the original HiFi reads and WGS reads via Merqury^9^. To advance the assembly to the chromosome level, we employed a reference-guided scaffolding strategy based on synteny with a closely related species, the Amur ratsnake (*Elaphe schrenckii*), which shares an identical karyotype (2n = 36)^10^. The contigs were systematically anchored and oriented into chromosome-scale scaffolds by using quarTeT (v 1.2.5)^11^, leveraging the high-quality chromosome-level genome of *Elaphe schrenckii*(GCA_050231175.1) as a reference. This approach resulted in the final assembly, designated ElaCar_v1. The genome size is 1.62 Gb with a contig N50 of 2.77 Mb and a scaffold N50 of 143.07 Mb (Fig. 1, Table 1). Approximately 90.75% (1.5 Gb) of the assembled sequences were successfully assigned to 18 pseudochromosomes, consistent with the species’ known diploid number. BUSCO assessment yielded a score of 97.3% completeness by using vertebrata_odb10^12^, indicating high genome integrity and quality (Fig. 2).

**Table 1:** *E. carinata* genome assembly statistics.

Features	Value
Assembly size (Gb)	1.62
Contig N50 (Mb)	2.77
Scaffold N50 (Mb)	143,07
Number of chromosomes	18
GC content (%)	40.44
BUSCO completeness (%)	97.3

### Repetitive sequence annotation

Repetitive elements were identified and annotated using Earl Grey v1.0, a reproducible pipeline that integrates de novo repeat discovery with homology-based masking^13^. The tool was installed via Bioconda, which automatically configures dependent software including RepeatModeler2^14^ and RepeatMasker^15^. The Dfam database (v3.7)^16^ was used as the primary reference library for repeat classification.

Repetitive sequences constituted 53.19% of the assembled genome, in which Long Interspersed Nuclear Elements (LINEs) represented the most abundant class of transposable elements, comprising 23.02% of the genome, followed by DNA transposons (6.18%) and Unclassified repeats (12.67%). LTR retrotransposons covered 3.60% of the genome, while SINEs and Penelope-like elements accounted for 2.62% and 1.22%, respectively. Additionally, simple repeats, microsatellites, and low-complexity sequences collectively represented 3.79% of the genomic sequence (Table 2).

**Table 2:** Classification of repetitive sequence in ***E.carinata*** genome.

Type	Repeat length (bp)	% of genome
DNA	99,982,391	6.2
Rolling Circle	1,457,683	0.1
Penelope	19,763,587	1.2
LINE	372,464,172	23.0
SINE	42,426,395	2.6
LTR	58,259,199	3.6
Other (Simple Repeat, Microsatellite, RNA)	61,303,748	3.8
Unclassified	204,949,000	12.7
Total	780,606,175	53.2

### Gene structure annotation

The annotation of protein-coding genes was performed by integrating evidence from ab initio prediction, homology-based inference, and transcriptomic data. The repeat-softmasked genome was used as the input. For homology-based prediction, protein sequences from five sauropsid species (*Ahaetulla prasina*^17^, *Anolis carolinensis*^18^, *Pantherophis guttatus*^19^, *Podarcis muralis*^20^, and *Python bivittatus*^21^) were aligned to the genome using GeMoMa (v1.7.1)^22^. Ab initio gene prediction was conducted with the BRAKER pipeline (v2.1.6)^23^, which integrated RNA-seq data to train AUGUSTUS^24^ and GeneMark-ETP^25^. Finally, all lines of evidence were integrated to generate a consensus gene set using EvidenceModeler (EVM, v2.0.0)^26^.

We predicted 19,750 high-confidence protein-coding genes in the *E. carinata* genome (Table 3). These gene models had an average gene length of 32,064 bp and an average CDS length of 1,587 bp (Table 3). BUSCO assessment of the annotated gene set showed a completeness of 93.3% (Fig. 2).

**Table 3:** *E. carinata* genome gene annotation summary.

Gene structure annotation	ElaCar_v1
Total number of genes	19,750
Total number of exons	179,889
Total number of introns	160,139
Average mRNA length (bp)	32,064
Average CDS length per gene (bp)	1,587
Average exon number per gene	9
Average exon length (bp)	234
Average intron length (bp)	3,692

### Gene function annotation

Functional annotation of protein-coding genes was performed using eggNOG-mapper v2^27^. The tool provided comprehensive annotations from multiple databases, including Gene Ontology (GO) terms, KEGG pathways, EC numbers, COG functional categories. Protein domain predictions were obtained by integrating PFAM and SMART annotations through the built-in homology-based pipeline. Overall, 99.2% of the predicted genes were successfully functionally annotated (Table 4 and S1).

**Table 4:** Functional annotation statistics of *E. carinata* genome.

Database	Number	Percent(%)
GO	15,004	81.1%
KEGG	13,896	75.2%
EC	3,665	19.8%
COG	18,320	99.1%
Total	18,344	99.2%

## Data Records

The PacBio HiFi long-reads and whole-genome sequencing (WGS) reads also have been deposited in the National Genomics Data Center (NGDC)^28,29^ under accession number PRJCA048610. Additionally, the HiFi genome sequencing data are accessible under accession number CRR2223119^30^, the whole-genome sequencing (WGS) data under CRR2223121^31^, and the transcriptome data under CRR2223120^32^. The complete genome sequence has been deposited in the NGDC Genome Warehouse under accession number GWHGZIJ00000000.1^33^. The PacBio HiFi sequencing data, RNA sequencing data, and Illumina whole-genome sequencing data have been also deposited in the National Center for Biotechnology Information (NCBI) SRA database under accession numbers SRR38272852^34^, SRR38272850^35^, and SRR38272851^36^. The genome assembly has been deposited at NCBI under the Whole Genome Shotgun project accession JBXVYC000000000^37^. The corresponding genome annotation is available via Figshare^38^.

## Technical Validation

Genomic DNA quality and quantity were assessed using a NanoDrop spectrophotometer and a Qubit fluorometer. For the next-generation sequencing (NGS) libraries, the Qubit-measured concentration was 58 ng/μL. For PacBio HiFi library preparation, the DNA concentration was 82 ng/μL, yielding a total mass of approximately 12.3 μg. Purity assessment showed A260/A280 ratios of 1.85 and A260/A230 ratios of 2.10 for both samples, indicating minimal contamination by proteins, phenol, or chaotropic salts. The ratio of NanoDrop-derived concentration to Qubit-derived concentration (Nc/Qc) was 1.2.

Genome assembly completeness and base-level accuracy were rigorously assessed. Benchmarking with BUSCO v5.4.4 against the vertebrata_odb10 database (n = 3,354) revealed 97.3% complete, 1.0% fragmented, and 1.7% missing BUSCOs. Additionally, the assembly achieved a quality value (QV) of 58.965, corresponding to an error rate of 1.27 × 10^−6^. These results collectively demonstrate that the assembled genome is highly complete and accurate, rendering it suitable for downstream comparative and functional genomic analyses.

## Supplementary information

Supplementary Information

## Data Availability

No custom scripts were generated for this work. All data processing and analyses were performed using publicly available software as described in the Methods section, with specific versions and parameters detailed therein.
